# Tributyltin induces conjugation of ATG8s to single membranes via the V-ATPase–ATG16L1 axis, leading to transcription factor EB activation in human cell lines

**DOI:** 10.1007/s00204-026-04300-7

**Published:** 2026-02-07

**Authors:** Shunichi Hatamiya, Masatsugu Miyara, Nanako Takahashi, Ami Oguro, Yaichiro Kotake

**Affiliations:** Graduate School of Biomedical and Health Sciences, Hiroshima University, Hiroshima, 734-8553 Japan

**Keywords:** Tributyltin, CASM, Noncanonical autophagy, Lysosome, LC3, TFEB

## Abstract

Tributyltin (TBT) is an environmental contaminant that induces diverse toxic effects in mammals, but the cellular mechanisms underlying adaptation to TBT stress remain poorly understood. Conjugation of ATG8s to single membranes (CASM) is a noncanonical LC3‑lipidation pathway activated by various stressors, distinct from canonical autophagy. We previously showed that TBT reduces lysosomal acidity and inhibits autophagy in SH-SY5Y cells. Furthermore, we observed TBT-induced LC3-II accumulation, which was reduced by bafilomycin A_1_, and tubular LC3-positive structures as hallmarks of CASM. In this study, we investigated whether TBT activates CASM. TBT (700 nM) induced LC3-II accumulation, which was completely blocked by bafilomycin A_1_ in SH-SY5Y and HeLa cells. Unlike autophagy, TBT induced LC3-II accumulation even under class III PI3K inhibition by wortmannin and in FIP200-knockout cells. Salmonella effector protein SopF, which inhibits V-ATPase**–**ATG16L1 association required for CASM, inhibited TBT-induced LC3-II accumulation. In FIP200-knockout cells, TBT induced LC3 accumulation on lysosomes, the primary CASM target. TBT also promoted nuclear translocation of transcription factor EB (TFEB) in a SopF-sensitive manner. Together, these results identify CASM as a lysosomal stress response to TBT, induced via the V-ATPase**–**ATG16L1 axis, leading to TFEB activation. This mechanism provides a toxicological framework for understanding xenobiotic-induced lysosomal adaptations.

**Supplementary Information:**

The online version contains supplementary material available at 10.1007/s00204-026-04300-7.

## Introduction

Tributyltin (TBT) is an environmental pollutant historically used in agricultural pesticides, antifouling paints, and polyvinyl chloride stabilizers (Kotake 2012). While its use has been banned in many countries, TBT persists in marine sediments because of slow degradation under anoxic and poorly mixed conditions (Turk et al. 2024). TBT is well known to cause adverse effects in marine animals, such as inducing imposex in marine gastropods (e.g. *Nucella lapillus*) (Evans et al. 1996). In mammals, TBT exposure causes diverse toxicities: rats show reduced motor activity and impaired avoidance behavior (Ema et al. 1991), whereas mice develop obesity, hepatic steatosis (Zuo et al. 2011), dermatitis-like lesions (Ohtaki et al. 2007), and immune dysfunction (Ueno et al. 2009; Im et al. 2015). However, the primary molecular targets and central mechanisms of TBT toxicity in mammals remain unclear.

Cells elicit stress response mechanisms upon TBT exposure. TBT affects multiple pathways, such as acting as an agonist of retinoid X receptor (RXR) (Grün et al. 2006) and peroxisome proliferator-activated receptor γ (Kanayama et al. 2005), downregulating RXR in hepatocytes (Stossi et al. 2019), impairing immune function by reducing granzyme B and perforin in natural killer cells (Thomas et al. 2004), inhibiting ATP synthesis (von Ballmoos et al. 2004), and decreasing AMPA-type glutamate receptor subunit GluA2 in neurons (Nakatsu et al. 2009) through persistent inhibition of nuclear respiratory factor-1 (Ishida et al. 2017). Furthermore, TBT induces oxidative stress, increases reactive oxygen species (ROS) production, and activates stress kinases such as JNK (Liu et al. 2008), which can lead to apoptosis (Huang et al. 2018). TBT exposure activates Keap1–Nrf2 pathway, which protects cells from various stimuli such as oxidative stress, via a reduction in Kelch-like ECH-associated protein 1 (Keap1) (Hatano et al. 2023). TBT also disrupts endoplasmic reticulum Ca^2^⁺ homeostasis, triggering an unfolded protein response characterized by markers such as GRP78 and CHOP (Isomura et al. 2013). Despite these findings, the broader cellular stress responses and adaptive mechanisms that maintain homeostasis during TBT exposure remain underexplored.

Autophagy and the conjugation of ATG8s to single membranes (CASM), both of which depend on microtubule-associated protein 1 light chain 3 (LC3) lipidation, play key roles in protein quality control and stress adaptation. Autophagy deficiency leads to neurodegeneration and liver tumorigenesis (Hara et al. 2006; Takamura et al. 2011). Autophagy is typically activated under nutrient starvation and proceeds through a well-defined sequence of steps (Suzuki et al. 2017). The autophagy initiation complex (ATG13, FIP200, ULK1/2) forms the initial membrane, followed by the class III PI3K complex (VPS34, VPS15, Beclin 1, ATG14L), which generates PI3P to recruit downstream proteins. During membrane maturation, LC3 is conjugated to phosphatidylethanolamine via the ATG12–ATG5–ATG16L1 complex, converting the cytosolic form (LC3-I) into the membrane-associated form (LC3-II). LC3-II-modified autophagosomes fuse with lysosomes to degrade their contents. LC3 is widely used as a marker of autophagosomes. CASM is a noncanonical pathway, where LC3 is conjugated to single membranes (Durgan and Florey 2022). Unlike autophagy, CASM does not require an autophagy initiation complex and is triggered by diverse stimuli including infections, ROS, and lysosomotropic agents. These stimuli promote V-ATPase V1–V0 assembly on lysosomal membranes, enabling ATG16L1 recruitment via its WD40 domain (Fletcher et al. 2018), which drives LC3 conjugation. CASM leads to LC3 localization on lysosomal membranes, usually as vesicles, although LC3-positive tubular lysosomes can occasionally be observed (Cross et al. 2023). CASM is functionally linked to transcription factor EB (TFEB) activation, a key regulator of lysosomal biogenesis (Goodwin et al. 2021).

Our previous work (Hatamiya et al. 2022) showed that TBT increases LC3-II levels without inducing cell death in SH-SY5Y cells. This effect was accompanied by lysosomal deacidification and impaired autophagic degradation, leading to LC3-II accumulation. Bafilomycin A_1_ (Baf), which typically increases LC3-II levels by inhibiting autophagic degradation, reduced LC3-II levels in TBT-treated cells—an established hallmark of CASM (Durgan and Florey 2022; Cross et al. 2023). We also observed tubular LC3-positive structures. Together, these findings suggest that TBT triggers CASM while simultaneously inhibiting autophagic degradation. However, whether CASM is a bona fide response to TBT and its physiological significance remain unknown. In this study, we aimed to investigate whether TBT activates CASM and explore its role in cellular stress adaptation.

## Materials and methods

### Reagents

TBT (T0363) was purchased from Tokyo Chemical Industry. Baf (11038) was purchased from Cayman Chemical. Wortmannin (W-2990) was purchased from LC Laboratories. Dimethyl sulfoxide (DMSO; 043-07216), NaCl (191-01665), Na_3_VO_4_ (198-09752), polyvinylidene difluoride (PVDF) membranes (033-23433), digitonin (043-21376), and MgCl_2_∙6H_2_O (135-00165) were purchased from FUJIFILM Wako Pure Chemical Corporation. TBT, Baf, and wortmannin were diluted with DMSO. Final DMSO concentration was 0.1%. Dulbecco’s Modified Eagle Medium (DMEM; 08456), penicillin and streptomycin solution (09367), Nonidet P-40 (23640-94), Tris–HCl (35406-91), EDTA (15105-35), Chemi-lumi One (07880, 02230, and 11644), 4% paraformaldehyde in phosphate buffer (09154), bovine serum albumin (BSA; 01863-48), Tryptone (35640-95), Yeast Extract Dried (15838-45), KCl (28538-75), glucose (09588-15), Luria Broth (LB) Agar, Miller (20069-65), and kanamycin monosulfate (08976-84) were purchased from Nacalai Tesque. NaF (450022) was purchased from Sigma-Aldrich. Fetal bovine serum (FBS; 175012) was purchased from NICHIREI BIOSCIENCES. Horse serum was purchased from Gibco-BRL. 4′,6-diamidino-2-phenylindole dihydrochloride (DAPI; D1306), ProLong Diamond Antifade (P36961), Lipofectamine LTX Reagent with PLUS Reagent (15338100), and Opti-MEM Reduced Serum Medium (31985070) were purchased from Thermo Fisher Scientific. pmCherry-C1 (632524), pAcGFP-C1 (632470), and E. coli DH5α Competent Cells (9057) were purchased from Takara Bio. XhoI (312-00392) and BamHI (315-00061) were purchased from NIPPON GENE. FastGene Gel/PCR Extraction Kit (FG-91302) was purchased from Nippon Genetics. Ligation High Ver. 2 (LGK-201) was purchased from TOYOBO. MgSO_4_ (25035-00) was purchased from KANTO CHEMICAL. FavorPrep Plasmid DNA Extraction Mini Kit (FAPDE001-1) was purchased from Favorgen Biotech Corporation.

### Antibodies

Antibodies used in this study are listed below. Anti-LC3 (PM036, WB 1:5000, IF 1:200) was purchased from MBL. Anti-vinculin (sc-73614, WB 1:10000), anti-LAMP1 (sc-20011, IF 1:100), anti-GFP (sc-9996, IF 1:200), and anti-Lamin B1 (sc-377000, WB 1:1000) were purchased from Santa Cruz Biotechnology. Anti-mCherry (26765-1-AP, WB 1:5000) was purchased from Proteintech. Anti-GM130 (AB_398142, IF 1:200) was purchased from BD Biosciences. Anti-TFEB (4240, WB 1:1000, IF 1:200) was purchased from Cell Signaling Technology. Horseradish peroxidase (HRP)-conjugated anti-mouse IgG (A9044, WB 1:5000) and HRP-conjugated anti-rabbit IgG (A9169, WB 1:5000) were purchased from Sigma-Aldrich. Alexa Fluor 488-conjugated goat anti-mouse IgG (A‑11001, IF 1:800) and Alexa Fluor 555-conjugated goat anti-rabbit IgG (A-21428, IF 1:800) were purchased from Thermo Fisher Scientific.

### Cell culture

Human neuronal SH-SY5Y cells (American Type Culture Collection, Manassas, VA, USA) were cultured in DMEM with 5% FBS, 5% horse serum, 1% penicillin and streptomycin. Wild-type HeLa cells [RCB5692, Riken BRC (Okawa et al. 2021; Chino et al. 2019; Tsuboyama et al. 2016; Tamura et al. 2017)] and FIP200-knockout (KO) HeLa cells [RCB5696, RIKEN RCB (Tsuboyama et al. 2016)] were cultured in DMEM containing 10% FBS, 1% penicillin, and streptomycin. All cells were cultured in incubators at 37 °C with 5% CO_2_.

### Cell lysis

Cell lysis was performed using method 1, as previously described (Miyara et al. 2016a), with minor modifications. Briefly, the cells were washed twice with PBS(-) and scraped into lysis buffer [1% Nonidet P-40, 20 mM Tris–HCl (pH 7.4), 150 mM NaCl, 2 mM EDTA, 50 mM NaF, and 1 mM Na_3_VO_4_]. Lysates were mixed for 30 min at 4 °C, then centrifuged at 13,500 rpm for 15 min. Supernatants were mixed with sample buffer and boiled.

### Western blotting

Western blotting was performed as previously described (Miyara et al. 2016b; Hatamiya et al. 2022) with minor modifications. Briefly, the samples were separated on 10% or 15% polyacrylamide SDS-PAGE gels and transferred to PVDF membranes. Membranes were blocked with blocking buffer (TBS-T containing 3–5% non-fat dry milk) and incubated with primary antibody diluted with blocking buffer at 4 °C overnight. Membranes were washed with TBS-T three times and incubated with HRP-conjugated secondary antibody diluted with blocking buffer at RT for 1 h. Membranes were washed three times and then developed with Chemi-lumi One. Images were captured using a luminescent image analyzer (FUSION SOLO.7S. EDGE, Vilber Lourmat). Band intensities were quantified via densitometric analysis using Evolution-Capt software (Vilber Lourmat).

### Immunofluorescence

Immunofluorescence was performed according to a previously described method (Miyara et al. 2016b) with minor modifications. Briefly, cells were fixed with 4% paraformaldehyde in phosphate buffer for 10 min. Samples were permeabilized with 0.1% digitonin in PBS(-) at RT for 5 min, and incubated with BSA/PBS [filtered 1% BSA in PBS(-)] at RT for 1 h. After washing with PBS(-) three times, the samples were incubated with the primary antibody in BSA/PBS at 4 °C overnight with agitation. Samples were washed three times with PBS(-) and then incubated with Alexa Fluor 488- or 555-conjugated secondary antibodies at RT for 1 h. After being washed with PBS(-), samples were stained with DAPI at RT for 5 min. Samples were mounted using ProLong Diamond Antifade. Images were captured with an Opera Phenix automated confocal microscope (Revvity) and processed using Harmony software (Revvity).

### Plasmid transfection

Cells were seeded on 35 mm and 60 mm dishes. pmCherry-C1, pmCherry-SopF [a gift from Leigh Knodler (Addgene plasmid # 135174)], and pAcGFP-SopF vectors were mixed with Lipofectamine LTX Reagent with PLUS Reagent in Opti-MEM Reduced Serum Medium. After incubation for 20 min at RT, DNA plasmids and lipid complexes were added to dishes or wells of the microplates. The medium was replaced with fresh medium 3 h after transfection, and cells were incubated at 37 °C with 5% CO_2_. Approximately 21 h later, the medium was replaced with fresh medium containing chemical reagents. For immunofluorescence, 75 ng plasmid DNA, 75 nL PLUS reagent, and 150 nL LTX reagent were added to each well of 96-well microplate. In other cases, 2 μg DNA plasmids, 2 μL PLUS reagent and 4 μL LTX reagent were used.

### Plasmid construction

The SopF coding fragment was excised from mCherry-SopF by double digestion with XhoI and BamHI and gel-purified using a FastGene Gel/PCR Extraction Kit according to the manufacturer’s instructions. The pAcGFP-C1 vector was linearized by double digestion with XhoI and BamHI, and gel-purified using the same kit. The purified insert and vector were ligated using Ligation High Ver. 2 following the manufacturer’s instructions. E. coli DH5α Competent Cells were transformed with the ligation product according to the manufacturer’s instructions and incubated in Super Optimal broth with Catabolite repression medium (8.6 mM NaCl, 2% tryptone, 0.5% Yeast Extract Dried, 2.5 mM KCl, 10 mM MgSO_4_, 10 mM MgCl_2_, 20 mM glucose) at 37 °C for 30 min with shaking at 250 rpm. The cells were then plated on LB agar plates containing 50 µg/mL kanamycin and incubated overnight at 37 °C. A single colony was inoculated into 2 × Yeast Extract Tryptone medium (1.6% tryptone, 1% Yeast Extract Dried, and 0.5% NaCl; pH 7) containing 50 µg/mL kanamycin and cultured overnight at 37 °C with shaking at 250 rpm. The plasmid construct was purified using the FavorPrep Plasmid DNA Extraction Mini Kit, according to the manufacturer’s instructions.

### Statistical analysis

Statistical analysis was performed using Mini-StatMate software (ATMS). The indicated P-values were obtained using one-way analysis of variance and Tukey’s post-hoc test, as indicated in the figure legends. P < 0.05 was considered significant.

## Results

### TBT induces CASM via the V-ATPase–ATG16L1 axis

**Fig. 1:**
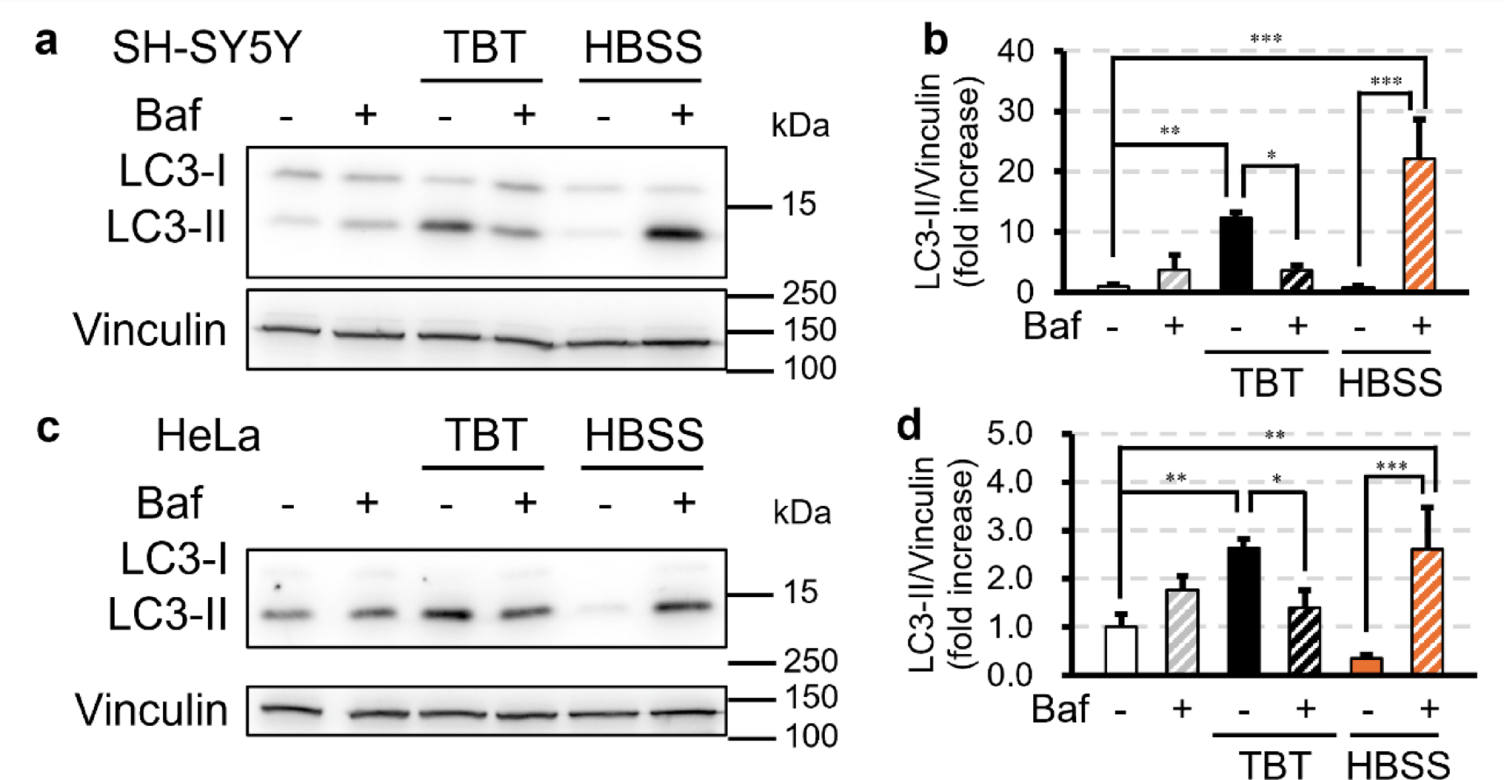
TBT increases LC3-II protein expression levels in a V-ATPase–dependent manner. **a**–**d** SH-SY5Y (**a**, **b**) and HeLa cells (**c**, **d**) were treated with 700 nM TBT or HBSS with or without 200 nM bafilomycin A_1_ (Baf) for 1 h. All samples in this figure were probed for LC3 and vinculin by Western blotting. Data are presented as mean + S.D. from three independent experiments. * P < 0.05, ** P < 0.01, *** P < 0.001 (Tukey test)

**Fig. 2:**
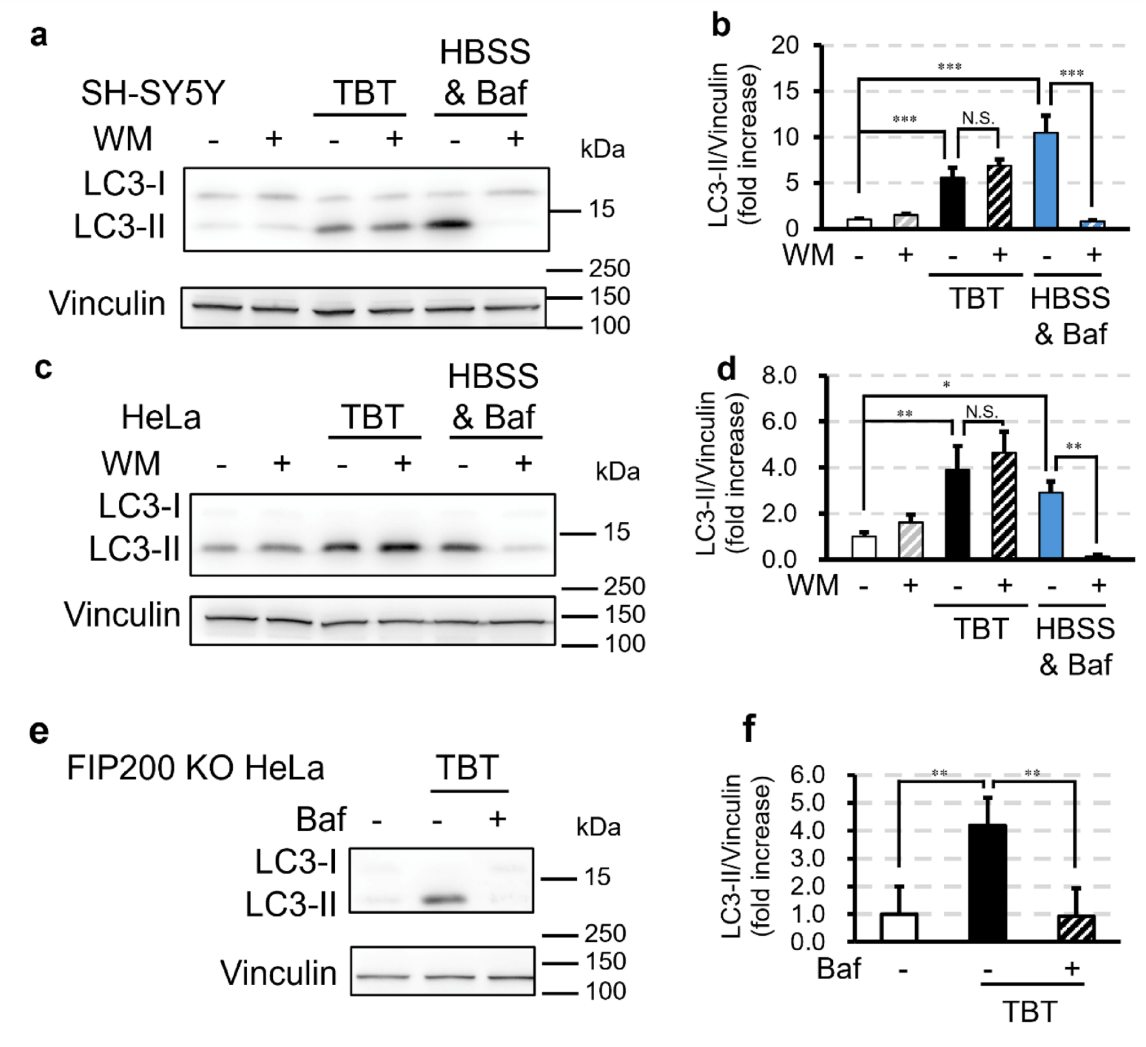
TBT induces LC3-II accumulation through CASM. **a**–**d** SH-SY5Y (**a**, **b**) and HeLa cells (**c**, **d**) were treated with 700 nM TBT or HBSS containing 200 nM bafilomycin A_1_ (Baf) with or without 500 nM wortmannin (WM) for 1 h. **e**, **f** FIP200-knockout (KO) HeLa cells were treated with 700 nM TBT with or without 200 nM Baf for 1 h. All samples in this figure were probed for LC3 and vinculin by Western blotting. Data are presented as mean + S.D. from three or four independent experiments. * P < 0.05, ** P < 0.01, *** P < 0.001 (Tukey test)

Autophagy requires an autophagy initiation complex (including FIP200) and PI3K complex, whereas CASM proceeds independently of these complexes. CASM depends on V-ATPase V1–V0 assembly (Hooper et al. 2022), while autophagy is independent of V-ATPase. In our recent study, treatment with 700 nM TBT for 6 h increased LC3-II protein expression without causing cell death, whereas 1 µM TBT induced cell death. Therefore, we used 700 nM TBT in this study to elicit robust CASM signals while avoiding overt cytotoxicity during short-term exposure. To confirm our recent observation that TBT-induced LC3-II accumulation is Baf-sensitive, SH-SY5Y cells were treated with 700 nM TBT with or without 200 nM Baf for 1 h. For comparison, we also used HBSS (amino acid-free, with Ca^2+^ and Mg^2+^; starvation medium), which induces autophagy, with or without Baf for 1 h. HBSS alone did not increase LC3-II, likely reflecting high basal autophagic flux (Klionsky et al. 2021), whereas HBSS + Baf markedly increased LC3-II, as expected for canonical autophagy (Fig. 1a, b). In contrast, TBT increased LC3-II, and this increase was suppressed by Baf—consistent with CASM activation (Fig. 1a, b). Similar results were observed in HeLa cells (Fig. 1c, d). To further test whether this LC3-II accumulation was CASM-dependent, SH-SY5Y cells were treated with 700 nM TBT with or without 500 nM wortmannin, a potent PI3K inhibitor. Wortmannin had no effect on TBT-induced LC3-II accumulation (Fig. 2a, b), while it strongly suppressed LC3-II accumulation in HBSS + Baf-treated cells, confirming PI3K inhibition. Similar results were obtained for HeLa cells (Fig. 2c, d). Finally, in FIP200 KO HeLa cells, TBT still increased LC3-II levels, and this increase was completely blocked by Baf (Fig. 2e, f). Together, these results demonstrate that TBT induces LC3-II accumulation through CASM.

**Fig. 3:**
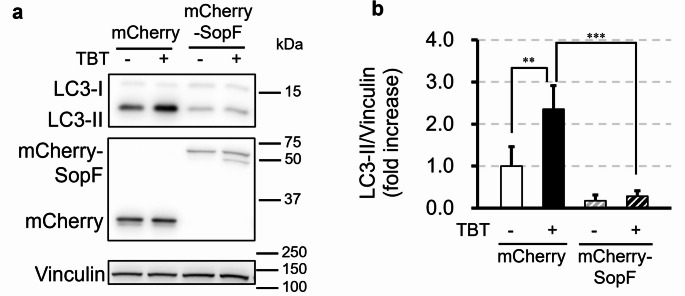
TBT-induced CASM depends on the V-ATPase–ATG16L1 association. **a**, **b** FIP200-knockout (KO) HeLa cells transiently expressing mCherry or mCherry-SopF were treated with 700 nM TBT for 1 h. All samples in this figure were probed for LC3, mCherry, and vinculin by Western blotting. Data are presented as mean + S.D. from three independent experiments. ** P < 0.01, *** P < 0.001 (Tukey test)

CASM is driven by the interaction between V-ATPase and ATG16L1, a process specifically inhibited by SopF, a *Salmonella* effector protein. SopF modifies the ATP6V0C subunit of V-ATPase via ADP ribosylation, thereby preventing its association with ATG16L1 (Hooper et al. 2022). To test whether TBT-induced CASM activation depends on the V-ATPase–ATG16L1 axis, we used FIP200 KO HeLa cells transiently expressing mCherry-SopF. Control cells expressing mCherry alone exhibited a significant increase in LC3-II protein expression after treatment with 700 nM TBT for 1 h (Fig. 3a, b). In contrast, cells expressing mCherry-SopF did not accumulate LC3-II after TBT exposure (Fig. 3a, b). These results suggest that CASM activation by TBT requires V-ATPase–ATG16L1 interaction.

### TBT-induced CASM results in TFEB activation

**Fig. 4:**
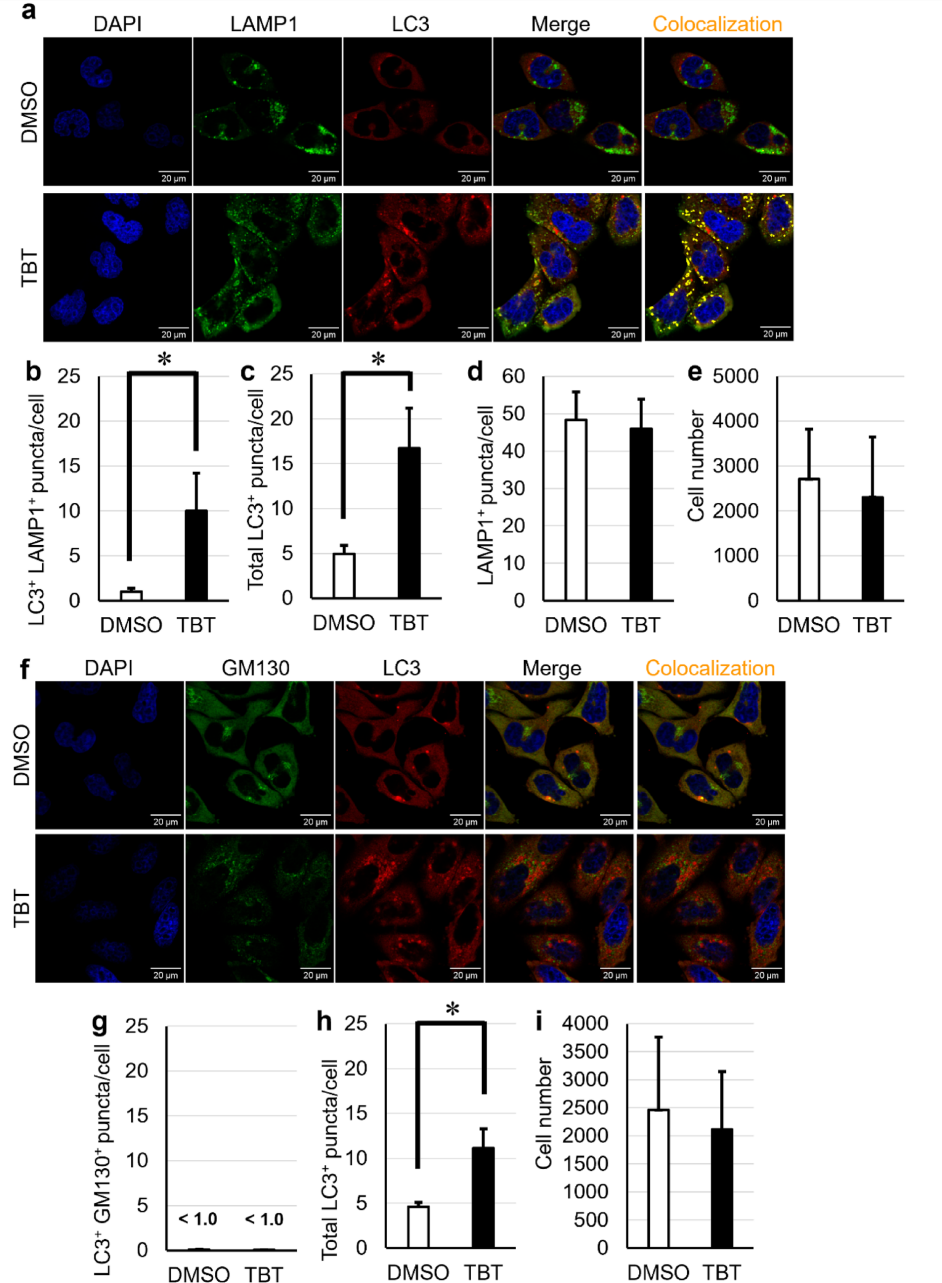
TBT triggers LC3 recruitment to lysosomes, but not to the Golgi apparatus. **a**–**e** FIP200-knockout (KO) HeLa cells were treated with 700 nM TBT for 6 h. Confocal images were stained for LC3 and LAMP1. Colocalized puncta (LC3+ and LAMP1+) are highlighted in yellow. Scale bars are 20 μm. **f–i** FIP200 KO HeLa cells were treated with 700 nM TBT for 6 h. Confocal images were stained for LC3 and GM130 (cis-Golgi marker). Colocalized puncta (LC3+ and GM130+) are highlighted in yellow. Scale bars are 20 μm. Data are presented as mean + S.D. from three independent experiments. * P < 0.05 (Tukey test)

CASM typically targets LC3 to endosomes or lysosomes, and in some cases the Golgi apparatus (Gao et al. 2016). To determine the subcellular localization of LC3 upon TBT exposure, we analyzed FIP200 KO HeLa cells treated with 700 nM TBT for 6 h using a high-content imaging system and analysis software to quantify LC3-positive puncta and organelle markers per cell. TBT significantly increased LC3-positive puncta, which largely colocalized with LAMP1, a lysosomal marker (Fig. 4a–e), but not with GM130, a Golgi marker (Fig. 4f–i), indicating lysosomal localization.

**Fig. 5:**
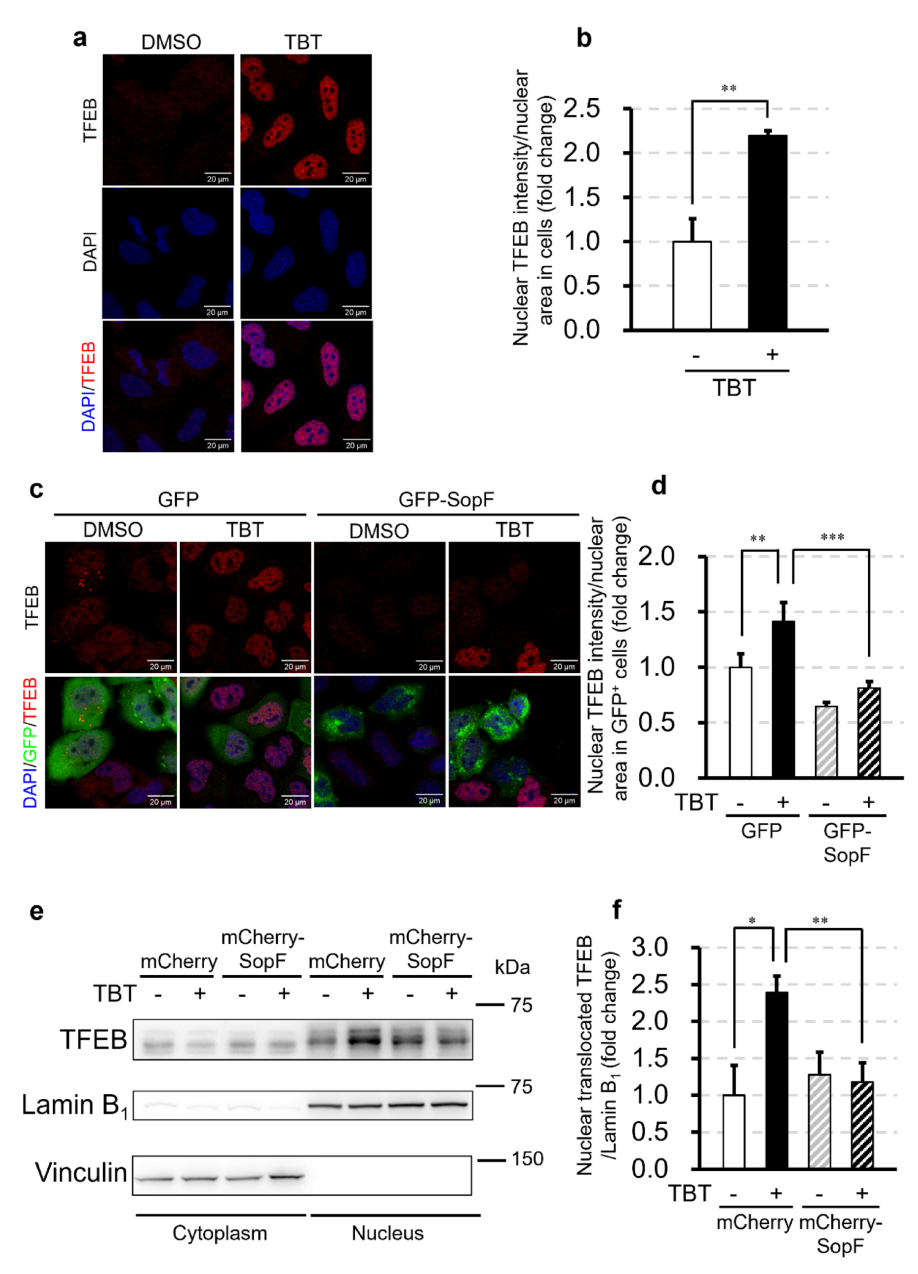
TBT-induced TFEB nuclear translocation requires V-ATPase**–**ATG16L1 association. **a**–**d** FIP200-knockout (KO) HeLa cells were treated with 700 nM TBT for 3 h. (**a**, **b**) Cells without exogenous expression; (**c**, **d**) cells transiently expressing GFP or GFP-SopF. Confocal images were stained for TFEB and, where applicable, GFP. ‘Nuclear TFEB’ denotes the mean nuclear TFEB signal intensity normalized to nuclear area on a per‑cell basis. Scale bars are 20 μm. **e**, **f** FIP200 KO HeLa cells transiently expressing mCherry or mCherry-SopF were treated with 700 nM TBT for 3 h, fractionated into cytoplasmic and nuclear fractions, and probed for TFEB, Lamin B_1_, and vinculin by Western blotting. Data are presented as mean + S.D. from three independent experiments. * P < 0.05, ** P < 0.01, *** P < 0.001 (Tukey test)

Lysosomal stress and CASM activation have been linked to TFEB nuclear translocation (Durgan and Florey 2022). Given that TBT disrupts lysosomal acidity and induces CASM, we tested whether it also activates TFEB. In FIP200 KO HeLa cells, 3 h treatment with 700 nM TBT induced TFEB nuclear translocation (Fig. 5a, b). To test CASM dependence, we transiently expressed GFP-SopF in FIP200 KO HeLa cells. While transiently expressed GFP alone did not affect TFEB localization, GFP-SopF expression completely abolished TBT-induced TFEB nuclear translocation (Fig. 5c, d).

To further confirm the CASM dependency of TFEB activation induced by TBT, FIP200 KO HeLa cells transiently expressing mCherry-SopF were fractionated into cytoplasmic and nuclear compartments, and TFEB protein expression levels were analyzed in each fraction. After 3 h of treatment with 700 nM TBT, TFEB levels in the nuclear fraction were significantly increased in mCherry-expressing cells, whereas 700 nM TBT did not affect nuclear TFEB levels in mCherry-SopF-expressing cells (Fig. 5e, f). These results demonstrate that TBT activates TFEB through CASM in a V-ATPase–ATG16L1–dependent manner.

## Discussion

In this study, we demonstrated that TBT activates CASM via the V-ATPase–ATG16L1 axis and promotes TFEB activation. Our previous work showed that TBT inhibits autophagic degradation by reducing lysosomal acidity; the present data extend this observation by revealing that lysosomal deacidification also triggers CASM. Several lines of evidence support this conclusion: (i) TBT-induced LC3-II accumulation was suppressed by Baf, but not by wortmannin, and was preserved in FIP200-deficient cells; (ii) SopF expression, which disrupts V-ATPase–ATG16L1 interactions, abolished LC3-II accumulation; and (iii) TBT recruited LC3 to lysosomes and promoted TFEB activation, both of which were SopF-sensitive. Collectively, these findings establish that TBT elicits a lysosomal stress response mediated by CASM, providing new mechanistic insight into its cellular toxicity.

Various lysosome-targeting agents, including ionophores and lysosomotropic compounds, have been reported to induce CASM. Among these, Saliphenylhalamide A (SaliP) inhibits V‑ATPase activity while stabilizing V1–V0 assembly, and it activates CASM with a concomitant decrease in lysosomal acidity (Hooper et al. 2022). TBT has been reported to bind the V0 sector and interacts non-covalently with V1 in *Thermus thermophilus*, inhibiting proton permeability and ATPase activity (Takeda et al. 2009). These observations raise the possibility that TBT may similarly facilitate V1–V0 assembly through its action on V-ATPase, thereby inducing CASM, although it remains unclear whether the reported binding of TBT to V-ATPase also occurs in mammalian cells. In contrast, Baf and concanamycin A (ConA) inhibit CASM by binding to the V0c subunit and blocking V0 rotation, thereby halting proton translocation (Wang et al. 2021; Durgan and Florey 2022). Overall, these findings suggest that the specific mode of V-ATPase modulation determines whether CASM is induced or suppressed. Investigating how agents reported to affect V-ATPase influence CASM could provide further insight into V-ATPase-mediated regulation of this pathway.

TFEB activation represents a key adaptive mechanism that promotes lysosomal biogenesis and metabolic flexibility under stress. TFEB activation via CASM specifically requires membrane‑conjugated γ‑aminobutyric acid type A receptor–associated protein (GABARAP), rather than LC3, with GABARAP sequestering the FLCN–FNIP complex at lysosomes (Goodwin et al. 2021). Based on our SopF-sensitive results, we propose that TBT triggers GABARAP lipidation at lysosomes, promoting TFEB activation and driving lysosomal adaptation. While the physiological consequences of CASM-mediated TFEB activation remain poorly understood, prior studies suggest a protective role. TFEB mitigates kidney injury in a mouse model of calcium oxalate (CaOx) nephropathy, which is accompanied by lysosomal permeabilization, and this TFEB activation is dependent on LC3 lipidation (Nakamura et al. 2020). Given that L-Leucyl-L-Leucine methyl ester induces CASM through lysosomal permeabilization in human cell lines (Cross et al. 2023), TFEB activation in this context is likely mediated by CASM. Furthermore, several lysosomotropic agents have been shown to induce CASM in cultured cells, and local anesthetics can activate CASM in human skin (Jacquin et al. 2017). However, in vivo evidence remains sparse, and no study has systematically examined the role of CASM under xenobiotic stress.

In conclusion, our findings highlight a previously unappreciated mechanism by which TBT activates CASM and promotes TFEB-driven lysosomal adaptation. Future work should determine whether CASM activation represents a protective response to xenobiotic exposure in vivo and whether pharmacological modulation of CASM could mitigate TBT-induced tissue injury.

## Supplementary Information

Below is the link to the electronic supplementary material.

Supplementary Material 1

## Data Availability

All data generated or analyzed during this study are included in this published article and its supplementary information files.
