# Supplementary material for: Tributyltin induces conjugation of ATG8s to single membranes via the V-ATPase–ATG16L1 axis, leading to transcription factor EB activation in human cell lines
Source: Arch Toxicol. 2026 Feb 7;100(5):1941–51. doi: 10.1007/s00204-026-04300-7 (PMC13086666; doi:10.1007/s00204-026-04300-7)
Supplement: Supplementary file 1 — Supplementary Material 1 [file 204_2026_4300_MOESM1_ESM.pdf]

Archives of Toxicology

Supplementary Information

Tributyltin induces conjugation of ATG8s to single membranes via the V-ATPase–ATG16L1 axis, leading to transcription factor EB activation in human cell lines

Shunichi Hatamiya[1], Masatsugu Miyara[1,†], Nanako Takahashi[1], Ami Oguro[1], Yaichiro Kotake[1,†]

[1]Graduate School of Biomedical and Health Sciences, Hiroshima University, Hiroshima 734-8553, Japan

[†]Corresponding authors:
Masatsugu Miyara; e-mail address: miyara128@hiroshima-u.ac.jp
Yaichiro Kotake; e-mail address: yaichiro@hiroshima-u.ac.jp

Fig. S1

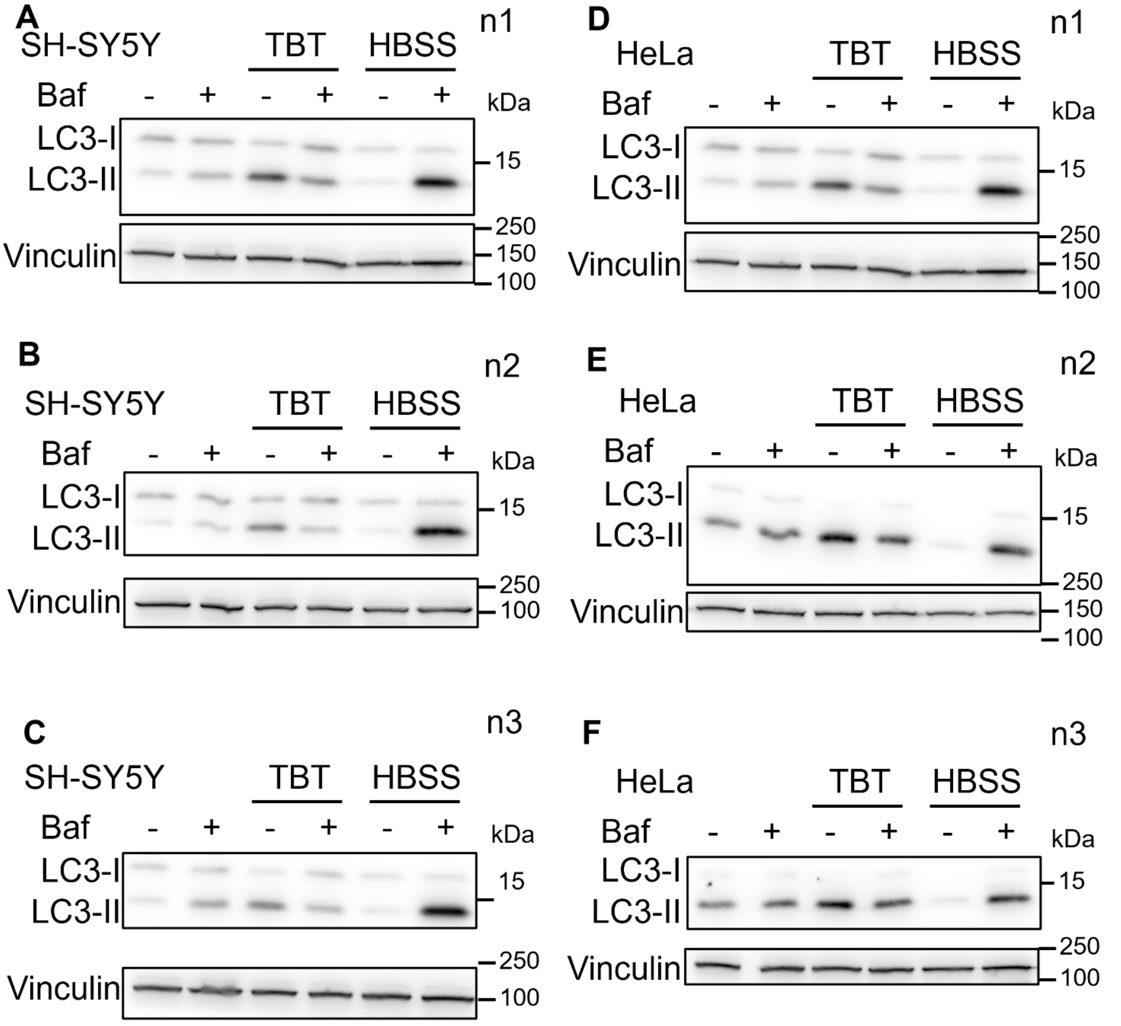

Fig. S1 Western blot images corresponding to Fig. 1a and 1c. **A–C** Three independent experiments corresponding to Fig. 1a. **D–F** Three independent experiments corresponding to Fig. 1c.

Fig. S2

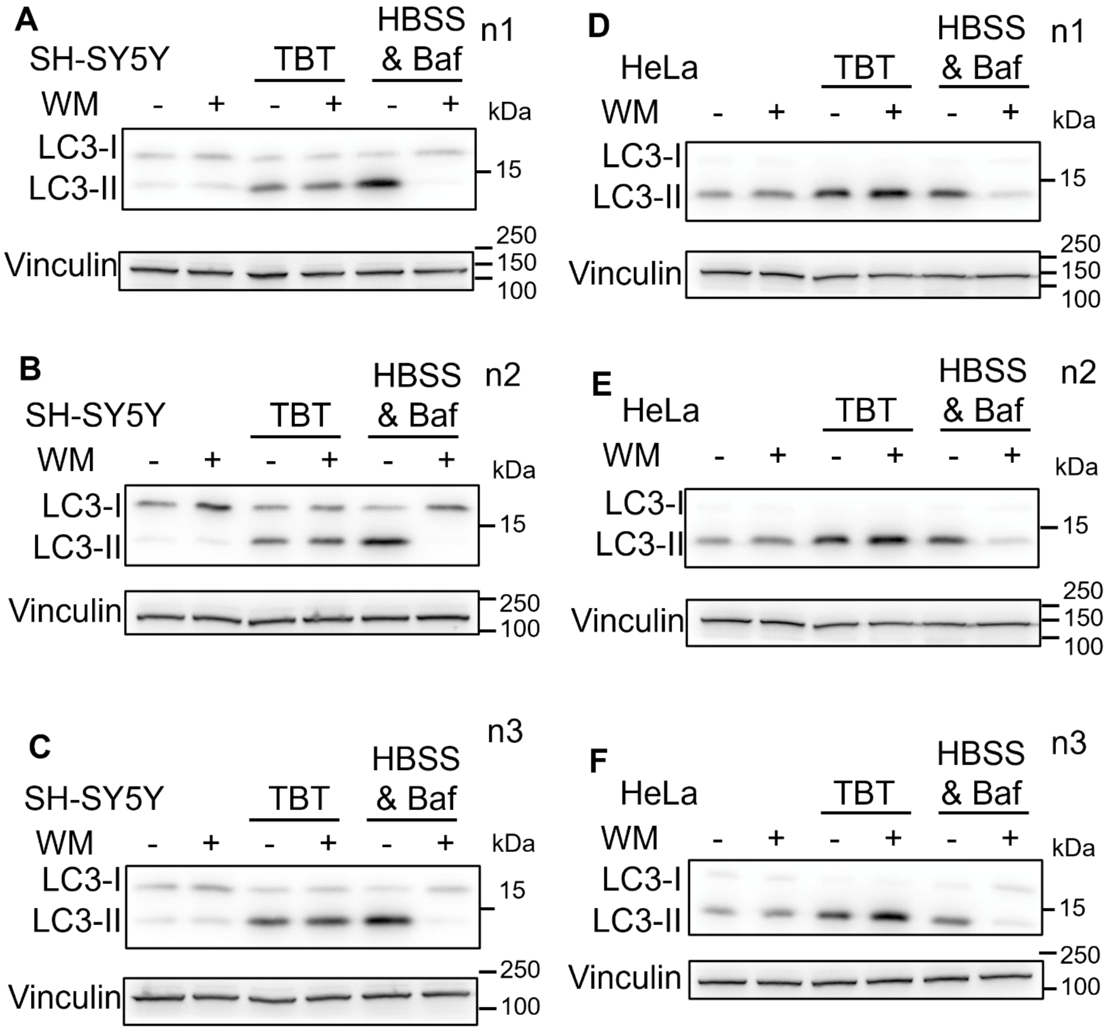

Fig. S2 Western blot images corresponding to Fig. 2a and 2c. **A–C** Three independent experiments corresponding to Fig. 2a. **D–F** Three independent experiments corresponding to Fig. 2c.

Fig. S3

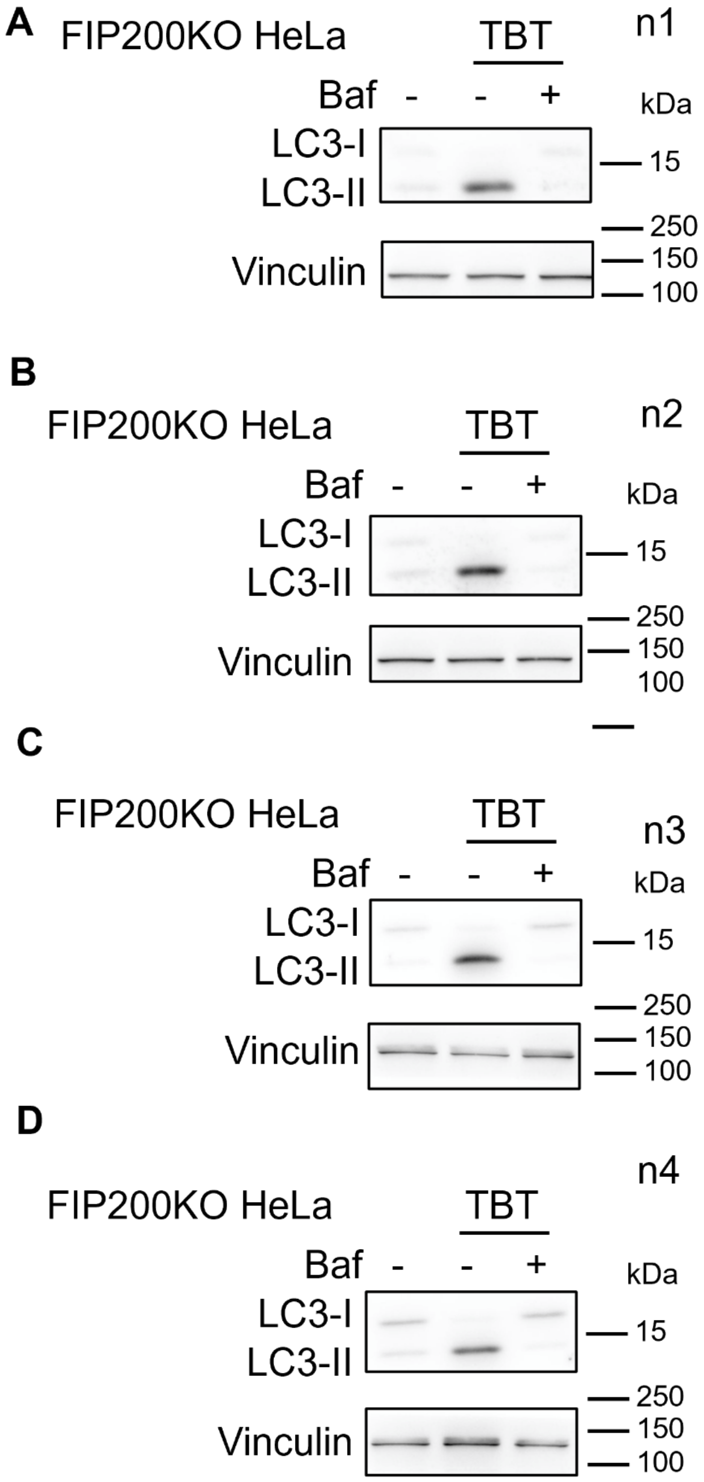

Fig. S3 Western blot images corresponding to Fig. 2e. **A–D** Four independent experiments corresponding to Fig. 2e.

Fig. S4

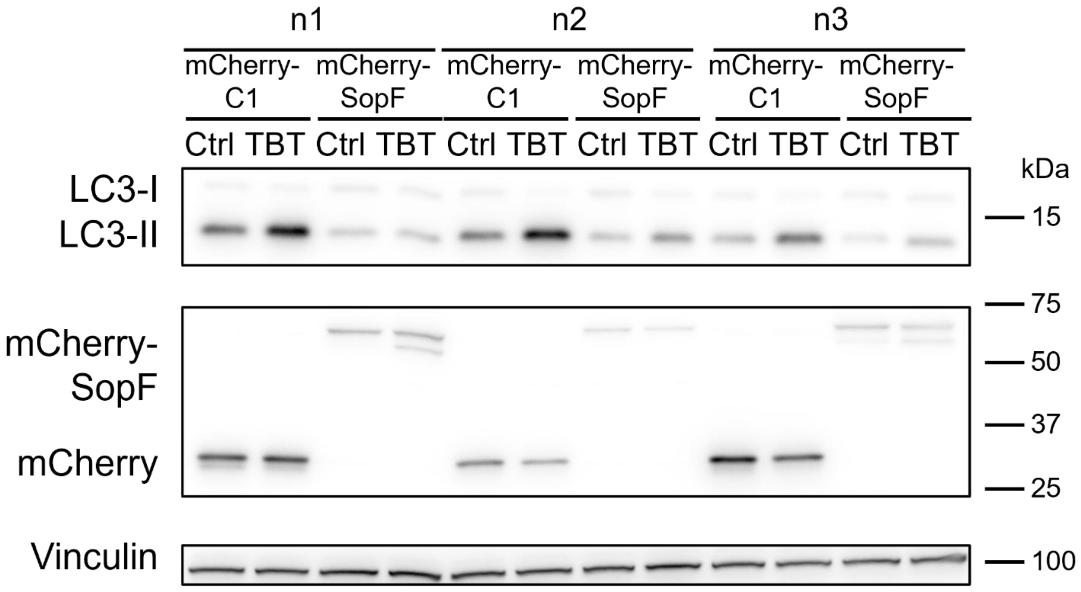

Fig. S4 Western blot images corresponding to Fig. 3a. Three independent experiments corresponding to Fig. 3a.

Fig. S5

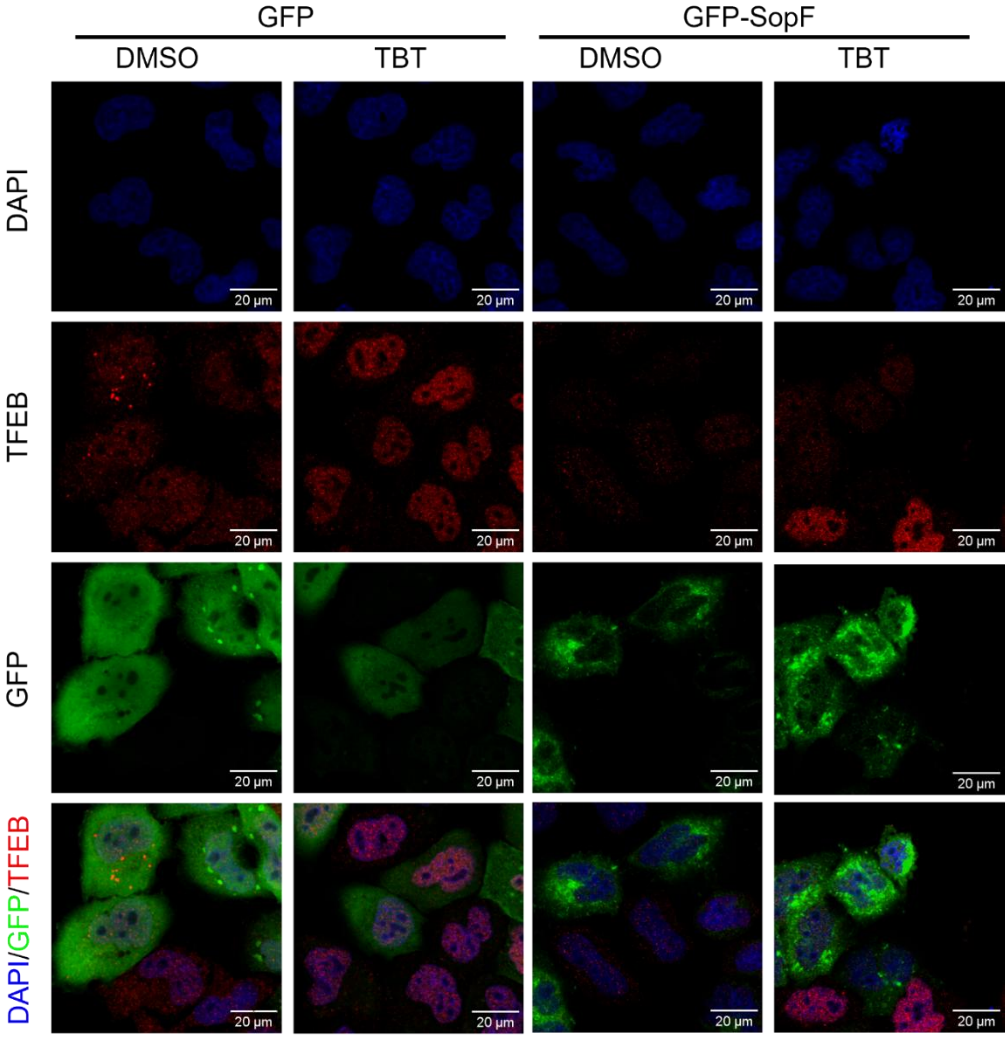

Fig. S5 Detailed confocal images corresponding to Fig. 5c.

Fig. S6

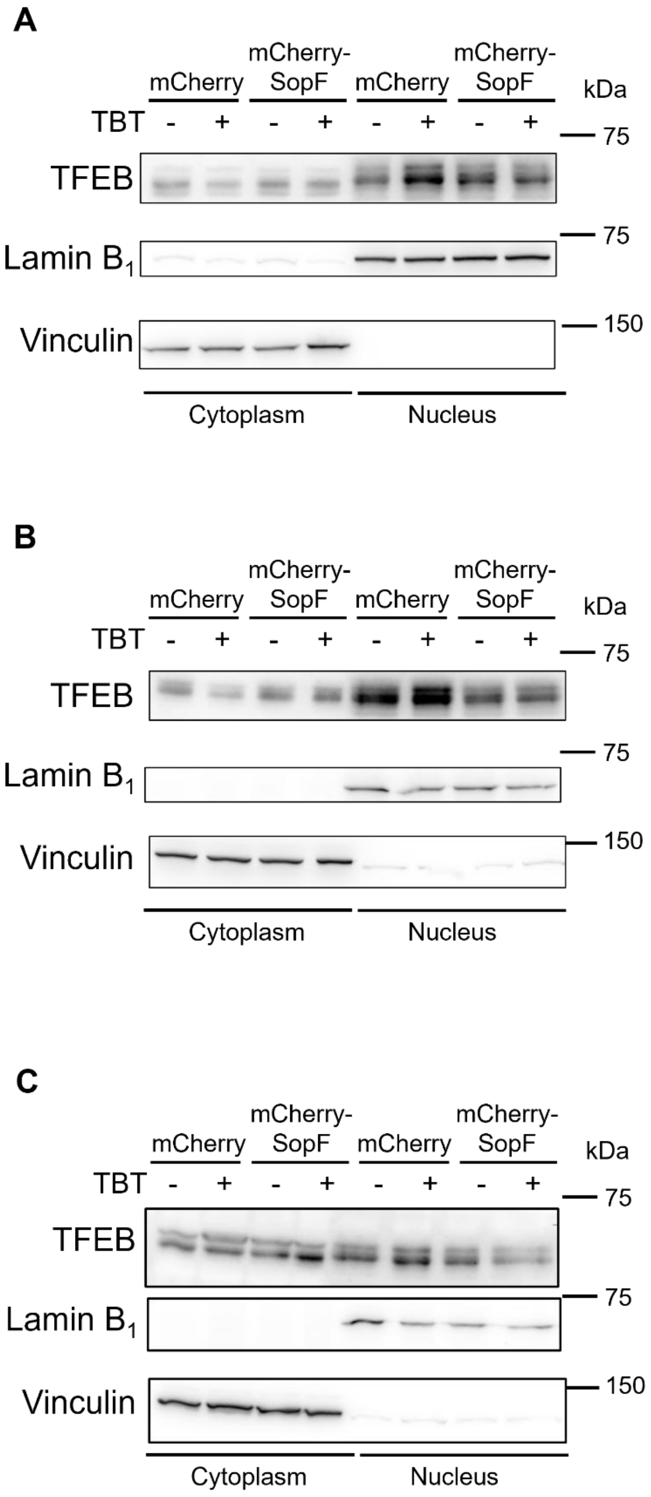

Fig. S6 Western blot images corresponding to Fig. 5e. **A–C** Three independent experiments corresponding to Fig. 5e.
